# High versus low ligation of the inferior mesenteric artery during transanal total mesorectal excision for low rectal cancer: impact on postoperative anastomotic leakage

**DOI:** 10.3389/fonc.2026.1881074

**Published:** 2026-07-16

**Authors:** Junfeng Du, Chen Su, Xiang Xu, Lanxin Hu, Xuefei Zhang, Hongyu Zhang

**Affiliations:** Department of Gastrointestinal Surgery, The First Affiliated Hospital of Chongqing Medical University, Chongqing, China

**Keywords:** anastomotic leakage, left colic artery preservation, low rectal cancer, surgical outcomes, transanal total mesorectal excision

## Abstract

**Introduction:**

Whether preserving the left colic artery (LCA) during transanal total mesorectal excision is beneficial remains unclear. This study evaluated the effects of LCA-preserving low ligation versus high ligation of the inferior mesenteric artery without LCA preservation on anastomotic leakage.

**Methods:**

We retrospectively reviewed patients with low rectal cancer at the First Affiliated Hospital of Chongqing Medical University (January 2014–January 2024). After propensity score matching, 182 patients were assigned to LCA preservation (n = 91, low ligation) or non-preservation (n = 91, high ligation) groups according to whether the LCA was preserved. Perioperative characteristics, pathological findings, and incidence of anastomotic leakage were compared, and subgroup analyses were performed.

**Results:**

The incidence of postoperative leakage in the LCA preservation group was lower than in the non-preservation group (4.3% vs. 13.2%, p = 0.036). Among patients who received neoadjuvant therapy, LCA preservation was associated with a lower incidence of anastomotic leakage rates (7.0% vs. 24.0%, p = 0.049).

**Discussion:**

LCA preservation may have clinical value in selected patients with low rectal cancer, particularly those receiving neoadjuvant therapy.

## 1 Introduction

Rectal cancer is one of the most common malignant tumors worldwide. With continuous advances in surgical techniques, treatment outcomes have improved substantially. Total mesorectal excision (TME) remains the standard surgical approach for rectal cancer. However, whether preservation of the left colic artery (LCA) during TME benefits patients remains debatable. As an emerging technique over the past decade, transanal TME (TaTME) has gradually become an important surgical option for specific patient populations (1), such as those with middle and low rectal cancers, particularly for patients with a difficult pelvis. By adopting a “bottom-up” approach, TaTME improves the visualization of the distal resection margin and enhances the integrity of the mesorectal specimen. Although TaTME was developed to overcome the technical challenges of traditional transabdominal TME performed deep within a narrow pelvis, anastomotic complications remain a major concern (2). These complications may be attributable to multiple factors, including patient-specific anatomical and physiological characteristics and surgical techniques.

In rectal cancer surgery, insufficient blood supply to the rectal anastomosis is considered a key risk factor for anastomotic leakage (3). The impact of preserving versus ligating the LCA on anastomotic perfusion has long been debated, particularly in TaTME, which is commonly performed for low rectal cancers located within 5 cm of the anal verge. Owing to the inherent anatomical challenges of these tumors, the risk of postoperative ischemia is higher than that in conventional mid- or high-rectal cancers (4). Yeh et al. (5) and Jestin et al. (6) reported that the risk of anastomotic leakage increased significantly when the distance between the anastomosis and the anal verge was less than 5–6 cm. During transanal surgery, the blood supply to the proximal anastomosis relies mainly on the marginal arterial arcade, whereas perfusion to the lower rectum is relatively poor. In addition, IMA dissection affects the main vascular trunk, and division of the LCA leaves a more limited blood supply, which may predispose the anastomosis to ischemia and leakage, particularly in the presence of tension or an inadequate marginal arch (7). In TaTME for low rectal cancer, the decision to preserve the LCA should therefore be made cautiously, provided oncological outcomes are not compromised (2–4). Although an increasing number of recent studies have examined the feasibility of LCA preservation and its potential effect on postoperative complications, the evidence remains controversial, with some studies supporting preservation (8–12), and others arguing against it (13–15). As an emerging technique, TaTME lacks clear guidance regarding LCA management.

Currently, no unified international guidelines or consensus exist on whether LCA should be preserved during TME. Guidelines from the National Comprehensive Cancer Network (NCCN) and the European Society for Medical Oncology (ESMO) do not explicitly recommend LCA preservation. The NCCN guidelines (16) advocate individualization based on patient and surgeon factors, whereas the ESMO guidelines (17) emphasize maintenance of blood supply. Meanwhile, the Chinese Society of Clinical Oncology guidelines (18) clearly recommend individualized consideration of LCA preservation.

Taken together, no unified consensus exists regarding preservation of the LCA during TME. Although TaTME has emerged as a novel surgical method, further research investigating the relationship between LCA preservation and anastomotic leakage in TaTME is lacking. Moreover, the benefits of selecting preservation of the LCA for patients remains unclear. Further research and clinical practice studies are crucial to clarify its feasibility and safety. Therefore, this study aimed to provide a scientific basis for individualized surgical decision-making by comparing and analyzing anastomotic leakage after TaTME with and without LCA preservation.

## 2 Materials and methods

### 2.1 Screening

This retrospective study was conducted in accordance with the principles of the Declaration of Helsinki and was approved by the Institutional Review Board of the First Affiliated Hospital of Chongqing Medical University (Approval No.: 2025-793-01). To protect patient privacy, all personal identifiers were removed, and participants were assigned numerical codes. No additional personal information was disclosed. Informed consent was obtained from all participants included in the study. All participants provided informed consent for publication of their anonymized clinical data included in this manuscript.

We enrolled consecutive patients with rectal cancer diagnosed via pathological biopsy, who were treated at the Department of Gastrointestinal Surgery, First Affiliated Hospital of Chongqing Medical University, from January 2014 to January 2024. Only patients with low rectal cancer, less than 5 cm from the anus, as determined using pelvic contrast-enhanced MRI, were included.

The inclusion criteria were as follows: (i) patients with biopsy-confirmed rectal adenocarcinoma requiring rectal cancer resection; (ii) no evidence of metastasis to other organs; and (iii) TNM stage I–III. The exclusion criteria were as follows: (i) distant organ or extensive peritoneal metastases before surgery; (ii) complications such as intestinal perforation, intestinal obstruction, or other emergencies; (iii) serious cardiopulmonary dysfunction; and (iv) substantial missing clinical data.

**Figure 1:**
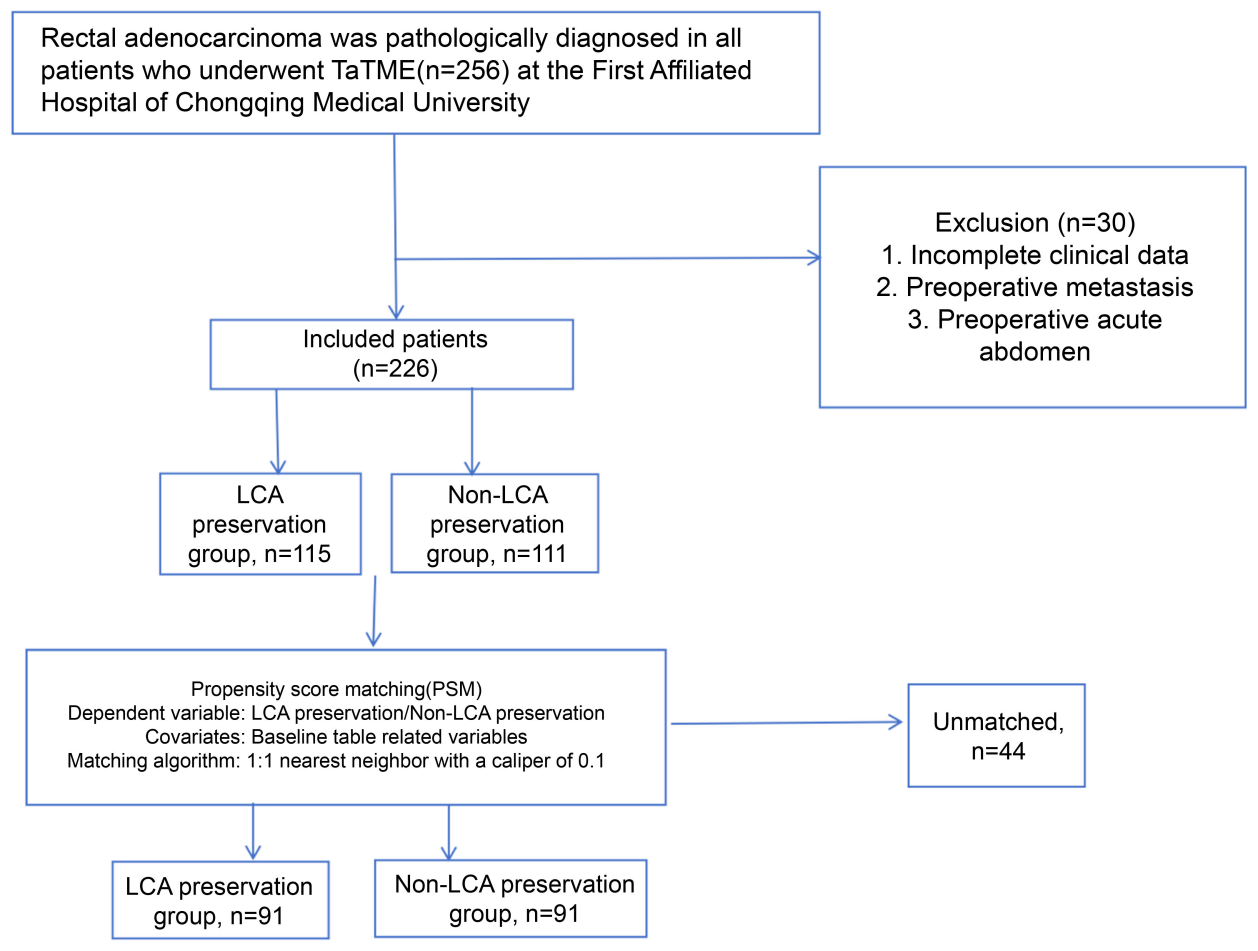
Flowchart of patient screening and selection. TaTME, transanal total mesorectal excision; LCA, left colic artery.

This single-center retrospective cohort study initially included 226 patients. After propensity score matching, 182 patients remained and were included in the final analysis (see **Figure 1** for details).

Before enrollment, the investigators explained the study’s objectives, significance, methodology, precautions, and related matters to all eligible patients, who independently decided whether to participate. All enrolled patients provided written informed consent.

### 2.2 Intraoperative methods

The TaTME procedure consisted of transabdominal and transanal components. All operations were performed by colorectal surgeons with more than 10 years of experience in TME surgery who belonged to the same surgical team and had completed the TaTME surgery learning curve.

All patients were placed in the lithotomy position and underwent surgery under general anesthesia with endotracheal intubation. Detailed operative procedures for TaTME in low rectal cancer are described in the Chinese guidelines (19). High ligation: the inferior mesenteric artery (IMA) was ligated at its origin from the aorta, and the LCA was routinely divided without preservation. Low ligation with LCA preservation: The IMA was ligated distal to the origin of the LCA and the continuity of the LCA and the marginal arteries was preserved. The decision to preserve or ligate the LCA was made by the surgeon based on the vascular anatomy, bowel length, anastomotic tension, and perfusion. LCA preservation was preferred when the collateral circulation (e.g., the Riolan arch) was absent or inadequate or the marginal arterial arch was poorly supplied, thereby increasing the risk of anastomotic ischemia, provided that bowel length allows for a tension-free anastomosis (with splenic flexure mobilization). Conversely, high ligation was performed when oncological radicality required ligation of the IMA at its origin or LCA trunk was involved by the tumor. These surgical principles were applied consistently across all the cases included throughout the 10-year study period. After completion of anastomosis, the pelvic cavity was irrigated, and an air leak test was performed. An anal drainage tube was selectively placed at the surgeon’s discretion based on intraoperative assessment of risk factors for anastomotic leakage, including inadequate blood supply, excessive tension, or edema at the anastomotic site. The decision to perform a diverting stoma was made by the surgeon based on the patient’s medical history and the condition of the anastomosis. Finally, a pelvic drain was placed and fixed to the patient’s abdominal wall.

### 2.3 Main outcome assessment

The primary outcome of this study was the occurrence of anastomotic leakage within 30 days after surgery. Anastomotic leakage was defined and graded according to the 2010 International Study Group of Rectal Cancer criteria. Grade A was defined as leakage requiring no active intervention; Grade B as leakage requiring non-surgical management, such as antibiotics or percutaneous drainage; and Grade C as leakage requiring reoperation. In this study, Grades B and C were classified as severe anastomotic leakage, whereas Grade A was classified as mild anastomotic leakage. Anastomotic leakage was diagnosed based on digital rectal examination, abdominal computed tomography, clinical manifestations, and surgical exploration, when necessary.

### 2.4 Statistical methods

Statistical analyses were performed using SPSS version 26.0 (IBM Corp., Armonk, NY, USA). Categorical variables were expressed as counts and percentages and were compared using the chi-square test when the sample size was sufficiently large and Fisher’s exact test when the sample size was small. Continuous variables were compared using the Mann–Whitney *U* test and are presented as medians with interquartile ranges. The number of lymph nodes harvested and the number of positive lymph nodes were analyzed using the t-test and are expressed as mean ± standard deviation. Multivariable logistic regression analysis was performed to identify independent predictors of postoperative complications. To minimize the potential influence of confounding factors, all variables were included in the multivariable logistic regression model using the forward likelihood ratio statistical method. Logistic regression results are reported as odds ratios (ORs) with 95% confidence intervals (CIs). A two-sided p value < 0.05 was considered statistically significant for all analyses.

## 3 Results

### 3.1 Patient characteristics

**Table 1** presents the participants’ clinical characteristics. After propensity score matching, the two groups demonstrated well-balanced baseline characteristics, with no statistically significant differences (all p > 0.05; **Table 2**; **Supplementary Table 1**).

**Table 1:** Clinical characteristics of patients with rectal cancer.

Characteristics	N = 182
Sex (n)	
Male	123 (67.6%)
Female	59 (32.4%)
Age (years)	59.50 (50.75, 67.00)
Body mass index (kg/m^2)^	22.49 (21.08, 24.41)
Diabetes mellitus(n)	17 (9.3%)
Smoking (n)	37 (20.3%)
Cardiomyopathy (n)	3 (1.6%)
Hyperlipidemia (n)	57 (31.3%)
Coronary heart disease (n)	10 (5.5%)
Mesenteric ischemic disease (n)	13 (7.1%)
Preoperative neoadjuvant therapy (n) Risk of anesthesia (n)	89 (48.9%)
II	148 (81.3%)
III	34 (18.7%)
Pelvic drainage tube placed (n)	172 (94.5%)
Anal drainage tube placed (n)	75 (41.2%)
Prophylactic stoma (n)	78 (42.9%)
Blood loss (mL)	100.00 (50.00, 165.00)
Albumin (g/L)	40.00 (38.00, 43.00)
LCA preserved with low ligation (n)	91 (50.0%)
Duration of surgery (min)	300.00 (255.00, 350.00)
Manual anastomosis (n) Pathological stage (n)	70 (38.5%)
I–II	138 (75.8%)
III	44 (24.2%)
Anastomotic leak (n)	16 (8.8%)

Variables are expressed as median (interquartile range) or n (%).

LCA, left colic artery.

**Table 2:** Preoperative baseline characteristics of the two groups.

Variable	LCA preserved with low ligation (n = 91)		LCA not preserved with high ligation (n = 91)	P-value
Sex (n)				
Male	63		60	0.750
Female	28		31	
Age (years)	58.000 (51.000, 67.000)		61.000 (50.000, 68.000)	0.510
Body mass index (kg/m2)	22.220 (20.760, 24.440)		22.830 (21.480, 24.410)	0.415
Diabetes mellitus (n)				
Yes	9		8	1.000
No	82		83	
Risk of anesthesia (n)				
II	74		74	1.000
III	17		17	
Smoking status (n)				
Smoking	17		20	0.713
Never smoked	74		71	
Cardiomyopathy (n)				
Yes	2		1	1.0000
No	89		90	
Albumin (g/L)	40.00 (38.00, 43.00)		40.00 (38.00, 42.00)	0.584
Hyperlipidemia (n)				
Yes	30		27	0.749
No	61		64	
Preoperative neoadjuvant therapy (n)				
Yes	43		46	0.767
No	48		45	
Pathological stage (n)				
I–II	67		71	0.604
III	24		20	
Coronary heart disease (n)				
Yes	6		4	0.747
No	85		87	
Mesenteric ischemic disease (n)				
Yes	7		6	1.000
No	84		85	
Pelvic drainage tube (n)				
Yes	86		90	0.211
No	5		1	
Anal drainage tube (n)				
Yes	38		37	1.000
No	53		54	
Prophylactic stoma (n)				
Yes	37		41	0.653
No	54		50	
Blood loss (mL)	100.0 (50.0, 200.0)		100.0 (50.0, 150.0)	0.483
Duration of surgery (min)	305.0 (260.0, 350.0)		300.0 (245.0, 350.0)	0.625
Anastomosis method (n)				
Manual	33		37	0.648
Non-manual	58		54	

Categorical variables are expressed as n and compared using the chi-square test. Continuous variables are presented as medians with interquartile ranges and compared using the Mann–Whitney *U* test. Statistical significance was set at p < 0.05.

LCA, left colic artery.

### 3.2 Overall analysis of postoperative complications

In the LCA preservation group, anastomotic leakage occurred in four patients; two cases were classified as grade C and two as grade B. In the non-preservation group, anastomotic leakage occurred in 12 patients. Ten patients had grade C anastomotic leakage, and 1 patient presented with a pelvic abscess. Two patients had grade B anastomotic leakage. A significant difference was observed in the incidence of anastomotic leakage between the LCA preservation and non-preservation groups (4.3% vs. 13.2%, p = 0.036, **Table 3**). Among these cases, 4 with grade B anastomotic leakage (2 cases each in the LCA preservation and LCA non-preservation groups) improved and were discharged after strict anti-infective therapy, parenteral nutritional support, and enhanced drainage. Twelve patients with grade C anastomotic leakage underwent abdominal irrigation and drainage combined with a diverting stoma in addition to the above treatments and were discharged after the leakage improved. The proportion of patients requiring surgical intervention for anastomotic leakage (12 cases of grade C leakage) also differed significantly between the groups (2.2% vs. 10.9%, p = 0.017). These findings suggest that preservation of LCA is associated with reduced severity of anastomotic leakage.

**Table 3:** Occurrence of anastomotic leakage.

	Occurrence (n)	Non-occurrence (n)	Total (n)	P-value
LCA preserved (n)	4	87	91	0.036
LCA not preserved (n)	12	79	91	
Total (n)	16	166	182	

Statistical significance was set at p < 0.05.

### 3.3 Logistic regression analysis

After propensity score matching, multivariable logistic regression analysis showed that hyperlipidemia, anal drainage tube placement and neoadjuvant therapy were independently associated with an increased risk of postoperative anastomotic leakage (**Table 4**). In contrast, LCA preservation was independently associated with a reduced risk of postoperative anastomotic leakage.

**Table 4:** Multivariate logistic regression analysis of postoperative complications.

Variable	Univariate analysis		P-value	Multivariate analysis		P-value
	OR 95% CI			OR 95% CI		
Age	1.008 0.960–1.058		0.762			
BMI (kg/m2)	0.993 0.887–1.112		0.902			
Sex	2.255 0.802–6.341		0.123			
Diabetes mellitus	2.505 0.637–9.855		0.189			
ASA	1.005 0.270–3.742		0.994			
Anesthesia risk						
Smoking	0.896 0.242–3.323		0.869			
Cardiomyopathy	0.000		0.999			
Pelvic drainage tube placed	0.000		0.999			
Anal drainage tube placed	**3.506 1.164–10.558**		**0.026**	**3.998 1.221–13.090**		**0.022**
Prophylactic stoma	0.783 0.272–2.256		0.651			
Blood loss (mL)	0.992 0.983–1.001		0.085			
Albumin (g/L)	1.073 0.948–1.215		0.265			
Hyperlipidemia	**3.161**	**1.114–8.971**	**0.031**	**3.572**	**1.138–11.206**	**0.029**
Preoperative neoadjuvant	**5.132**	**1.410–**	**0.013**	**4.073**	**1.066–**	**0.040**
therapy		**18.679**			**15.560**	
Duration	1.006	0.994–1.006	0.975			
of surgery (min)						
Hand-sewn anastomosis	0.706	0.235–2.126	0.536			
Pathological stage	2.021	0.690–5.921	0.200			
LCA	**0.303**	**0.094–0.977**	**0.046**	**0.274**	**0.079–0.948**	**0.041**
preserved						
Coronary heart disease	1.163	0.138–9.841	0.892			
Mesenteric ischemic disease	0.856	0.104–7.041	0.885			

Statistical significance was set at p < 0.05.

BMI, body mass index; OR, odds ratio; CI, confidence interval; ASA, American Society of Anesthesiologists; LCA, left colic artery 470.

Variables with statistical significance.

### 3.4 Subgroup analysis

#### 3.4.1 Preoperative neoadjuvant therapy-related subgroups

Among the patients who received preoperative neoadjuvant therapy, the overall rate of postoperative anastomotic leakage was lower in the LCA preservation group than in the non-preservation group (7.0% vs. 24.0%, p = 0.049; **Supplementary Table 3**). However, there was no significant difference in patients who did not receive preoperative neoadjuvant therapy (**Supplementary Table 4**).

#### 3.4.2 Hyperlipidemia-related subgroups

No significant difference was observed in the effect of hyperlipidemia on postoperative anastomotic leakage between the two groups (see **Supplementary Tables 5**, **6**).

#### 3.4.3 Anal drainage tube placement-related subgroups

No significant difference was observed in the effect of anal drainage tube placement on postoperative anastomotic leakage between the two groups (**Supplementary Tables 7**, **8**).

### 3.5 Lymph node dissection and prognosis analysis

Thirty-five patients were lost to follow-up because of their referral to other hospitals or refusal to review. Based on the data of 147 patients who were not lost to follow-up for 3 consecutive years, no significant difference was observed between the two groups in terms of the total number of lymph nodes obtained, number of positive lymph nodes, and positive rate of lymph nodes (**Supplementary Table 9**). Similar results were observed for disease-free survival at 3 years (85.7% vs. 84.3%, p = 0.808).

## 4 Discussion

Based on the results of this retrospective analysis, preservation of the LCA was associated with a lower risk of postoperative anastomotic leakage after TaTME (OR = 0.274, 95% CI: 0.079–0.948, p = 0.041). Preoperative neoadjuvant therapy (OR = 4.073, 95% CI: 1.066–15.560, p = 0.040), anal drainage tube placement (OR = 3.998, 95% CI: 1.221–13.090, p = 0.022), and hyperlipidemia (OR = 3.572, 95% CI: 1.138–11.206, p = 0.029) were identified as risk factors for postoperative anastomotic leakage following TaTME. Exploratory analyses further suggested that patients receiving neoadjuvant therapy before surgery may particularly benefit from LCA preservation. Diabetes was not included in the subgroup analysis after propensity score matching, because of the small number of patients with diabetes, resulting in insufficient statistical power. (**Supplementary Table 2**).

However, as this was a single-center retrospective cohort study, the subgroup sample size was relatively small. These findings indicate that LCA preservation may have potential clinical value in selected patient populations.

### 4.1 Ischemic mechanisms of TaTME surgery

As a novel surgical approach for rectal cancer, TaTME offers advantages, such as securing an adequate distal resection margin and overcoming the technical limitations imposed by a narrow pelvis. However, the risk of anastomotic ischemia and related complications remains a major challenge after TaTME (20). Although the bottom-up approach improves exposure of the deep pelvic cavity, it also results in early division of the middle rectal artery and the lateral branches of the median sacral artery. Compared with conventional transabdominal TME, TaTME disrupts these collateral vessels earlier and more extensively in the deepest part of the pelvis, where the operating field is limited, potentially weakening the collateral perfusion network of the distal rectal stump. Consequently, blood supply to the anastomosis becomes more dependent on perfusion from the proximal colon through the marginal arterial arcade via the middle colic artery, with additional contribution from the LCA when preserved, while the reserve blood supply to the distal stump is reduced. In the lower rectum, where TaTME is most commonly performed, collateral circulation is already relatively limited. This reduction in perfusion may be further exacerbated in patients with hyperlipidemia or a history of neoadjuvant therapy. During TaTME, prolonged placement of the transanal platform causes continuous mechanical compression of the blood vessels in the submucosal and muscular layers of the rectal wall. This may affect blood circulation (21). Furthermore, the operation is associated with a steep learning curve and demands high technical proficiency, both of which can influence the occurrence of postoperative complications. These anatomical issues suggest that preservation of LCA may help to improve blood supply to the anastomosis. Poor blood supply during TaTME is a contributor to anastomotic leakage, and LCA preservation may be associated with a reduced risk of anastomotic leakage. However, it must be acknowledged that these mechanistic explanations remain theoretical inferences based on surgical principles, as this study did not directly measure microcirculatory perfusion (e.g., indocyanine green fluorescence imaging) or assess blood supply and associated traction forces.

### 4.2 Vascular protective mechanisms of LCA preservation

LCA preservation maintains adequate blood supply to the proximal colon at the anastomotic site. When the proximal colon relies solely on collateral circulation from the middle colic artery, it may result in intestinal wall ischemia, edema, and reduced tensile strength at the anastomosis. Anastomotic tension is influenced by the movable length of the proximal colon and the integrity of the mesentery release. When the LCA is preserved, the ligation plane can be located at the distal end of the IMA after LCA separation (low ligation), eliminating the need to expose the IMA root. In TaTME, preservation of the LCA appears to help alleviate hemodynamic imbalance caused by collateral vessel separation and may ensure a more stable perfusion gradient. Although this approach might increase intraoperative traction stress, mobilizing the splenic flexure can prolong the colonic mobility and promote tension-free reconstruction, which is crucial in TaTME. The mobilization of the splenic region combined with LCA preservation allows the surgeon to optimize vascular continuity and potentially reduce the ischemic vulnerability inherent in the pelvic dissection. Simultaneously, the anatomical course of the LCA can serve as a reliable landmark to guide the medial-to-lateral dissection, which may increase the colon reach distance by 2–3 cm (22–25). These findings support considerations for preserving the LCA in patients with low rectal cancer undergoing TaTME. Further validation is needed through prospective randomized studies using standardized perfusion monitoring or matched cohorts in the future.

### 4.3 Precision insights from subgroup analysis

Poor glycemic control and hyperglycemia impair wound healing and increase susceptibility to infection, thus contributing to higher postoperative complication rates (26). Hyperglycemia disrupts inflammation-mediated responses, including vasodilation, bacterial opsonization, neutrophil adhesion, chemotaxis, and phagocytosis. These disruptions lead to reduced peripheral blood flow and impaired angiogenesis, ultimately delaying wound healing (27–30). Such immunologic and physiological disturbances can compromise the anastomosis, increase infectious complications, and worsen surgical outcomes. The results of multicenter and cohort studies show that patients with diabetes undergoing colorectal surgery have a significantly increased risk of postoperative surgical site infection (31–33) and anastomotic leakage (34, 35).

Yamano et al. (36) demonstrated that neoadjuvant chemoradiotherapy increases the risk of anastomotic leakage. Schiffmann et al. (37), in a study of 387 patients with rectal cancer, reported a postoperative leakage rate of 26.6% among patients receiving neoadjuvant therapy, compared with 9.7% among those who did not. Radiotherapy induces collagen deposition and suppresses cellular metabolism. When anastomotic perfusion relies predominantly on marginal vessels from the middle colic artery, perfusion may be inadequate. Furthermore, chemical drugs and radiation-related mucosal atrophy, submucosal vascular thickening, luminal occlusion, and perivascular fibrosis can cause local circulatory disturbances (38). Based on our study findings, we speculated that preservation of the LCA may be beneficial in reducing the incidence rate of anastomotic leakage in patients receiving neoadjuvant therapy (7.0% vs. 24.0%, p = 0.049). This observation is consistent with Teng’s hypothesis (39). These results suggest that LCA preservation may be a protective strategy worth considering in high-risk patients with low rectal cancer undergoing TaTME.

Regarding hyperlipidemia, no significant difference in the incidence of anastomotic leakage was noted between the two groups (10.0% vs 22.2%, p = 0.283). Recent research has shown that modulation of intestinal flora—particularly the ratio of Firmicutes and Bacteroidetes—can influence lipid metabolism (40). Hyperlipidemia may alter the intestinal flora and increase susceptibility to infection, thereby contributing to a higher risk of anastomotic leakage.

Regarding the anal drainage tube, although subgroup analyses did not reach statistical significance (**Supplementary Tables 7**, **8**), the observed association likely reflects a “marker of surgical concern” rather than a direct causal effect. In clinical practice, surgeons often selectively place anal drainage tubes selectively in patients perceived to have high-risk anastomoses (41). Notably, the incidence of anastomotic leakage was consistently lower in the LCA-preservation groups regardless of whether an anal drainage tube was placed, suggesting that the tube itself does not negate the effect of LCA preservation (42).

### 4.4 Surgical safety and technical optimization

In our study, no significant differences were observed in the operative time (305.0 (260.0, 350.0) vs. 300.0 (245.0, 350.0), p = 0.625) or intraoperative blood loss (100 (50.0, 200.0) vs. 100 (50.0, 150.0), p = 0.483) between the groups, reflecting the increased maturity and precision of contemporary anatomical preservation techniques.

Regarding lymph node dissection, no significant differences were observed between the two groups in the total number of lymph nodes retrieved (12.73 ± 2.927 vs. 13.79 ± 3.615, p = 0.052) and positive rate (0.081 ± 0.112 vs. 0.083 ± 0.097, p = 0.872), suggesting that LCA preservation may not affect lymph node dissection. Additionally, the proportion of hand-sewn anastomosis was lower in the preservation group (36.3% vs. 40.7%), It is suggested that preservation of LCA may be associated with improved feasibility of stapler anastomosis.

### 4.5 Dialectical interpretation of controversies and limitations

Our research also supports the ESMO guideline recommendation favoring optimal preservation of blood supply (17). Overall, these findings add to the growing body of evidence suggesting that LCA preservation improves anastomotic integrity. Our results also highlight the importance of individualized surgical strategies aligned with the principles of precision medicine.

However, this single-center retrospective cohort study has certain limitations. The subgroup sample sizes were small, and the limited number of adverse events resulted in wide CIs, indicating insufficient statistical power. Furthermore, the relatively short follow-up duration and limited oncologic survival data preclude definitive conclusions regarding the long-term benefits of LCA preservation. Although propensity score matching balanced the baseline characteristics, residual selection bias cannot be excluded because the decision to preserve the LCA was made at the surgeon’s discretion. Specifically, the vascular anatomical variation, tumor characteristics, technical difficulty, colon length, anastomotic tension, splenic flexure mobilization, and intraoperative perceived blood supply risk may all affect the choice of ligation level and postoperative outcome, and we could not completely exclude the interference of these confounding factors. In addition, we did not compare several variables, including anastomotic height, intraoperative perfusion, and air leak test results, surgical approach, conversion rate, and operator experience between groups. Furthermore, this was a single-center study conducted at a high-volume institution where the surgical team had already completed the TaTME learning curve. During the study period, treatment protocols, perioperative care, TaTME technique, and team experience may gradually change. Additionally, the learning curve is long, and the procedure is technically demanding, thus limiting generalizability to lower-volume hospitals or surgeons still navigating the TaTME learning curve. Finally, the proposed mechanisms remain theoretical, as we lacked the objective intraoperative data (e.g., indocyanine green fluorescence imaging) necessary to directly quantify microcirculatory changes.

In conclusion, this study suggests that LCA preservation was associated with a lower risk of postoperative Anastomotic leakage in TaTME for low rectal cancer. The preservation of LCA may have clinical value for specific low rectal cancer patients, especially those receiving neoadjuvant therapy. In the future, randomized, prospective multicenter studies with standardized perfusion assessments will be essential to further validate the clinical and oncologic safety of LCA preservation in TaTME.

## Data Availability

The raw data supporting the conclusions of this article will be made available by the authors, without undue reservation.
