# Supplementary material for: High versus low ligation of the inferior mesenteric artery during transanal total mesorectal excision for low rectal cancer: impact on postoperative anastomotic leakage
Source: Front Oncol. 2026 Jul 16;16:1881074. doi: 10.3389/fonc.2026.1881074 (PMC13422177; doi:10.3389/fonc.2026.1881074)
Supplement: Supplementary file 1 [file Table1.docx]

**Supplementary Table 1.** Preoperative baseline characteristics of the two groups before propensity-score matching

| **Variable** | **LCA preserved with low ligation (n = 115)** | **LCA not preserved with high ligation (n = 111)** | **p-value** |
| --- | --- | --- | --- |
| Sex (n) |  |  |  |
| Male | 82 | 74 | 0.475 |
| Female | 33 | 37 |  |
| Age (years) | 56.000 (50.0, 67.0) | 62.000 (51.0, 68.0) | 0.096 |
| Body mass index (kg/m2) | 22.230 (20.8, 24.4) | 22.830 (20.9, 24.4) | 0.415 |
| Diabetes mellitus (n) |  |  |  |
| Yes | 11 | 9 | 0.816 |
| No | 104 | 102 |  |
| Risk of anesthesia (n) |  |  | 0.259 |
| II | 87 | 91 |  |
| III | 28 | 20 |  |
| Smoking status (n) |  |  | 0.257 |
| Smoking | 19 | 26 |  |
| Never smoked | 96 | 85 |  |
| Cardiomyopathy (n)   \| Yes \| 2 \| 2 \| \| --- \| --- \| --- \| | | | 1.0000 |

| No | 113 | 109 |  |
| --- | --- | --- | --- |
| Albumin (g/L) | 40.00 (38.00, 42.00) | 40.00 (38.00, 43.00) | 0.399 |
| Hyperlipidemia (n) |  |  | 0.153 |
| Yes | 42 | 30 |  |
| No | 73 | 81 |  |
| Preoperative neoadjuvant therapy (n) |  |  | 0.424 |
| Yes | 50 | 55 |  |
| No | 65 | 56 |  |
| Pathological stage (n) |  |  | 0.280 |
| I–II | 84 | 88 |  |
| III | 31 | 23 |  |
| Coronary heart disease (n) |  |  | 1.0000 |
| Yes | 7 | 6 |  |
| No | 108 | 105 |  |
| Mesenteric ischemic disease (n) |  |  | 0.497 |
| Yes | 9 | 12 |  |
| No | 106 | 99 |  |
| Pelvic drainage tube (n) |  |  | 0.065 |
| Yes | 104 | 108 |  |
| No | 11 | 3 |  |

| Anal drainage tube (n) |  |  | 0.137 |
| --- | --- | --- | --- |
| Yes | 52 | 39 |  |
| No | 63 | 72 |  |
| Prophylactic stoma (n) |  |  | 0.103 |
| Yes | 40 | 56 |  |
| No | 75 | 55 |  |
| Blood loss (mL) | 100.0 (72.5, 200.0) | 100.0 (50.0, 150.0) | 0.096 |
| Duration of surgery (min) | 305.0 (260.0, 350.0) | 300.0 (245.0, 350.0) | 0.212 |
| Anastomosis method (n) |  |  | 0.055 |
| Manual | 36 | 49 |  |
| Non-manual | 79 | 62 |  |

Categorical variables are expressed as n and compared using the chi-square test. Continuous variables are presented as medians with interquartile ranges and compared using the Mann–Whitney *U* test. Statistical significance was set at p < 0.05.

LCA, left colic artery

**Supplementary Table 2.** Multivariate logistic regression analysis of postoperative complications before propensity-score matching

| **Variable** | **Univariate analysis** | | | **Multivariate analysis** | | |
| --- | --- | --- | --- | --- | --- | --- |
|  | **OR** | **95% CI** | **p-value** | **OR** | **95% CI** | **p-value** |
| Age | 1.012 | 0.968–1.057 | 0.608 |  |  |  |
| BMI (kg/m^2^) | 0.990 | 0.883–1.111 | 0.863 |  |  |  |
| Sex | 1.884 | 0.710-5.000 | 0.203 |  |  |  |
| Diabetes mellitus | **4.949** | **1.555–15.747** | **0.007** | **4.401** | **1.198–16.167** | **0.026** |
| ASA  Anesthesia risk | 1.065 | 0.334-3.397 | 0.915 |  |  |  |
| Smoking | 0.790 | 0.219–2.857 | 0.720 |  |  |  |
| Cardiomyopathy | 0.000 |  | 0.999 |  |  |  |
| Pelvic drainage tube placed | 0.000 |  | 0.999 |  |  |  |
| Anal drainage tube placed | 2.514 | 0.936–6.753 | 0.067 |  |  |  |
| Prophylactic stoma | 0.870 | 0.386–1.960 | 0.737 |  |  |  |
| Blood loss (mL) | 0.993 | 0.984–1.001 | 0.085 |  |  |  |
| Albumin (g/L) | 1.034 | 0.914–1.170 | 0.592 |  |  |  |
| Hyperlipidemia | **2.944** | 1.109–7.812 | **0.030** | **3.101** | 1.082–8.887 | **0.035** |
| Preoperative neoadjuvant therapy | **4.500** | 1.433–14.133 | **0.010** | **3.347** | 1.017–11.013 | **0.047** |
| Duration of surgery (min) | 1.000 | 0.994–1.006 | 0.926 |  |  |  |
| Manual anastomosis | 0.816 | 0.295–2.262 | 0.697 |  |  |  |
| Pathological stage | 1.667 | 0.594–4.676 | 0.332 |  |  |  |
| LCA preserved | **0.250** | 0.080–0.784 | **0.017** | **0.196** | 0.057–0.675 | **0.010** |
| Coronary heart disease | 0.961 | 0.118–7.841 | 0.970 |  |  |  |
| Mesenteric ischemic disease | 1.243 | 0.266–2.822 | 0.782 |  |  |  |

Statistical significance was set at p < 0.05.

BMI, body mass index; OR, odds ratio; CI, confidence interval; ASA, American Society of Anesthesiologists; LCA, left colic artery

**Supplementary Table 3.** Postoperative outcomes in patients receiving preoperative neoadjuvant therapy

|  | **Complications (n)** | **No complications (n)** | **Total (n)** | **p-value** |
| --- | --- | --- | --- | --- |
| LCA preserved (n) | 3 | 40 | 43 | 0.049 |
| LCA not preserved (n) | 10 | 36 | 46 |  |
| Total (n) | 13 | 76 | 89 |  |

Statistical significance was set at p < 0.05.

LCA, left colic artery

**Supplementary Table 4.** Postoperative outcomes in patients not receiving preoperative neoadjuvant therapy

|  | **Complications (n)** | **No complications (n)** | **Total (n)** | **p-value** |
| --- | --- | --- | --- | --- |
| LCA preserved (n) | 1 | 47 | 48 | 0.520 |
| LCA not preserved (n) | 2 | 43 | 45 |  |
| Total (n) | 3 | 90 | 93 |  |

Statistical significance was set at p < 0.05.

LCA, left colic artery

**Supplementary Table 5.** Postoperative outcomes in patients with hyperlipidemia

|  | **Complications (n)** | **No complications (n)** | **Total (n)** | **p-value** |
| --- | --- | --- | --- | --- |
| LCA preserved (n) | 3 | 27 | 30 | 0.283 |
| LCA not preserved (n) | 6 | 21 | 27 |  |
| Total (n) | 9 | 48 | 57 |  |

Statistical significance was set at p < 0.05.

LCA, left colic artery

**Supplementary Table 6.** Postoperative outcomes in patients without hyperlipidemia

|  | **Complications (n)** | **No complications (n)** | **Total (n)** | **p-value** |
| --- | --- | --- | --- | --- |
| LCA preserved (n) | 1 | 60 | 61 | 0.115 |
| LCA not preserved (n) | 6 | 58 | 64 |  |
| Total (n) | 7 | 118 | 125 |  |

Statistical significance was set at p < 0.05.

LCA, left colic artery

**Supplementary Table 7.** Postoperative outcomes in patients with anal canal placement

|  | **Complications (n)** | **No complications (n)** | **Total (n)** | **p-value** |
| --- | --- | --- | --- | --- |
| LCA preserved (n) | 3 | 35 | 38 | 0.113 |
| LCA not preserved (n) | 8 | 29 | 37 |  |
| Total (n) | 11 | 64 | 75 |  |

Statistical significance was set at p < 0.05.

LCA, left colic artery

**Supplementary Table 8.** Postoperative outcomes in patients without anal canal placement

|  | **Complications (n)** | **No complications (n)** | **Total (n)** | **p-value** |
| --- | --- | --- | --- | --- |
| LCA preserved (n) | 1 | 52 | 53 | 0.363 |
| LCA not preserved (n) | 4 | 50 | 54 |  |
| Total (n) | 5 | 102 | 107 |  |

Statistical significance was set at p < 0.05.

LCA, left colic artery

**Supplementary Table 9.** Lymph node dissection analysis

|  | **Total number of lymph nodes harvested (n)** | **Number of positive lymph nodes (n)** | **Lymph node positive rate** |
| --- | --- | --- | --- |
| LCA preserved (n) | 12.73 ± 2.927 | 0.91 ± 0.948 | 0.081 ± 0.112 |
| LCA not preserved (n) | 13.79 ± 3.615 | 1.16 ± 1.410 | 0.083 ± 0.097 |
| **p-value** | 0.052 | 0.209 | 0.872 |

Statistical significance was set at p < 0.05.

LCA, left colic artery
